# Case Report: Exploring KMT2D mutation in Shone syndrome

**DOI:** 10.3389/fcvm.2026.1651823

**Published:** 2026-04-23

**Authors:** Peiwen Cheng, Corlina Matthew, Guozhen Wang, Xia Xie, Yong An

**Affiliations:** 1 Department of Thoracic Surgery, Children's Hospital of Chongqing Medical University, National Clinical Research Center for Children and Adolescents' Health and Diseases, Ministry of Education Key Laboratory of Child Development and Disorders, Chongqing Key Laboratory of Structural Birth Defect and Reconstruction, Chongqing, China; 2 Department of Respiratory Medicine, Children's Hospital of Chongqing Medical University, National Clinical Research Center for Children and Adolescents' Health and Diseases, Ministry of Education Key Laboratory of Child Development and Disorders, Chongqing, China

**Keywords:** Kabuki syndrome, KMT2D, left-sided obstructive congenital heart disease, Shone complex, whole-exome sequencing

## Abstract

**Introduction:**

Shone syndrome is a rare congenital heart disease characterized by multilevel left-sided obstructive lesions. KMT2D variants cause Kabuki syndrome and are frequently associated with left-sided obstructive congenital heart defects, but their contribution to Shone syndrome remains uncertain.

**Methods:**

We report a 4-year-old girl with a Shone syndrome phenotype. Trio whole-exome sequencing (WES) was performed in the proband and her parents, with Sanger sequencing for confirmation and segregation analysis. Echocardiography and CT angiography were used to delineate cardiac anatomy.

**Results:**

Imaging and intraoperative findings demonstrated a supramitral ring with mitral stenosis, abnormal subvalvular apparatus, an interrupted/hypoplastic aortic arch with extensive collateral circulation, and a bicuspid aortic valve. Trio-WES identified a heterozygous KMT2D variant (NM_003482.4:c.15565G > A, p.Gly5189Arg) in the proband, which was also present in her clinically unaffected mother and younger sister, indicating maternal inheritance with variable expressivity.

**Conclusions:**

This report describes a child with multilevel left-sided obstruction and an inherited KMT2D variant, together with limited Kabuki-like facial features that did not meet the 2019 international consensus clinical criteria for Kabuki syndrome. While the observation raises the possibility that KMT2D-related pathways may contribute to Shone complex, a causal relationship cannot be inferred from a single family and requires additional genetic and functional studies.

## Introduction

Shone syndrome is a rare congenital cardiac disorder characterized by multilevel left-sided inflow and outflow obstruction. In the classic complete form described by Shone et al. in 1963, four obstructive lesions are present: a supravalvular mitral ring, parachute mitral valve, subaortic stenosis, and coarctation of the aorta (1). This constellation of anomalies is rare and typically presents in infancy with a cardiac murmur and signs of diminished systemic perfusion (e.g., feeding difficulties, tachypnea) due to left ventricular outflow obstruction (2). Partial (incomplete) forms involving two or three of the four classic lesions are also recognized, and additional cardiac anomalies such as a bicuspid aortic valve, patent ductus arteriosus, or septal defects may coexist in Shone syndrome (3).

Kabuki syndrome (KS) is an uncommon multisystem developmental disorder most often caused by autosomal dominant variants in KMT2D and less frequently by variants in KDM6A. Congenital heart defects are reported in approximately 50%–70% of individuals with KS, with a predominance of left-sided obstructive lesions such as coarctation of the aorta and other left ventricular outflow tract defects (4). Clinical studies suggest an association between KMT2D-related Kabuki syndrome and left-sided obstructive heart lesions (5). However, the genetic contribution of KMT2D variants to multilevel left-sided obstructive disease outside the classic KS phenotype remains incompletely understood. Here we report a child with a Shone syndrome phenotype and a rare, maternally inherited KMT2D missense variant, and we discuss the implications and limitations of this observation, including variable expressivity within the family.

## Methods

### Ethical statement

The study was approved by the Ethics Committee of Children's Hospital of Chongqing Medical University (File No.2025-434). All participants provided written informed consent in accordance with the 2013 revision of the Declaration of Helsinki and local regulations, covering surgery, genetic testing, and publication of anonymised clinical data and images (6).

### Study population and data collection

This single-case report involves a 4-year-old girl diagnosed with Shone syndrome in September 2024. Demographic, clinical, imaging, and operative data were extracted from the electronic medical record and independently reviewed by two paediatric cardiologists.

### Genetic analysis

The proband and her parents underwent trio whole-exome sequencing (WES) using the MyGenostics WES016-1-CQ V6 platform, with a mean sequencing depth >200×. Sequencing was performed on an Illumina NovaSeq platform. Reads were aligned to hg19 using BWA-MEM; variant calling was performed with GATK HaplotypeCaller and annotated with ANNOVAR. A heterozygous missense variant in KMT2D (NM_003482.4:c.15565G > A, p.Gly5189Arg; chr12:49420184, hg19) was identified in the proband. Sanger sequencing confirmed the variant and demonstrated maternal inheritance; the father was negative (7). According to the clinical laboratory report, this variant was classified as likely pathogenic based on PS4_Moderate, PM2_Supporting, and PP3_Strong evidence.

To assess possible alternative genetic explanations, we reviewed the broader trio-WES report rather than focusing on KMT2D alone. One additional phenotype-adjacent variant was identified in RELN (NM_005045.4:c.6149C > T, p.Thr2050Ile), inherited from the father and classified as a variant of uncertain significance (VUS). Because RELN is associated primarily with familial temporal lobe epilepsy and neuronal migration disorders, this finding may be more relevant to the proband's seizure phenotype than to her cardiovascular phenotype. The report also listed several other disease-associated variants, including ALPL c.18del (maternal, pathogenic), TSHR c.1349G > A (paternal, pathogenic), SLC3A1 c.163C > T (paternal, likely pathogenic), and ADAMTS2 c.1593dup (paternal, likely pathogenic). These findings were considered less compelling explanations for the proband's principal cardiovascular phenotype because their known disease spectra did not match the clinical presentation and/or the inheritance pattern did not support a stronger alternative diagnosis. Selected non-KMT2D findings are summarized in Supplementary Table S1.

## Case presentation

The proband is a 4-year-old girl born at term (approximately 40 weeks of gestation) to a mother with an obstetric history of gravida 3, para 3. The neonatal period was unremarkable. At 2 years of age, she began experiencing recurrent unprovoked focal seizures lasting approximately 1 min, followed by fatigue and somnolence. There was no loss of consciousness and no seizures during sleep. At 4 years of age, she was admitted for evaluation of complex congenital heart disease. Physical examination showed stable vital signs, no central cyanosis, a systolic murmur, and a blood pressure difference of approximately 50 mmHg between the upper and lower extremities.

**Figure 1:**
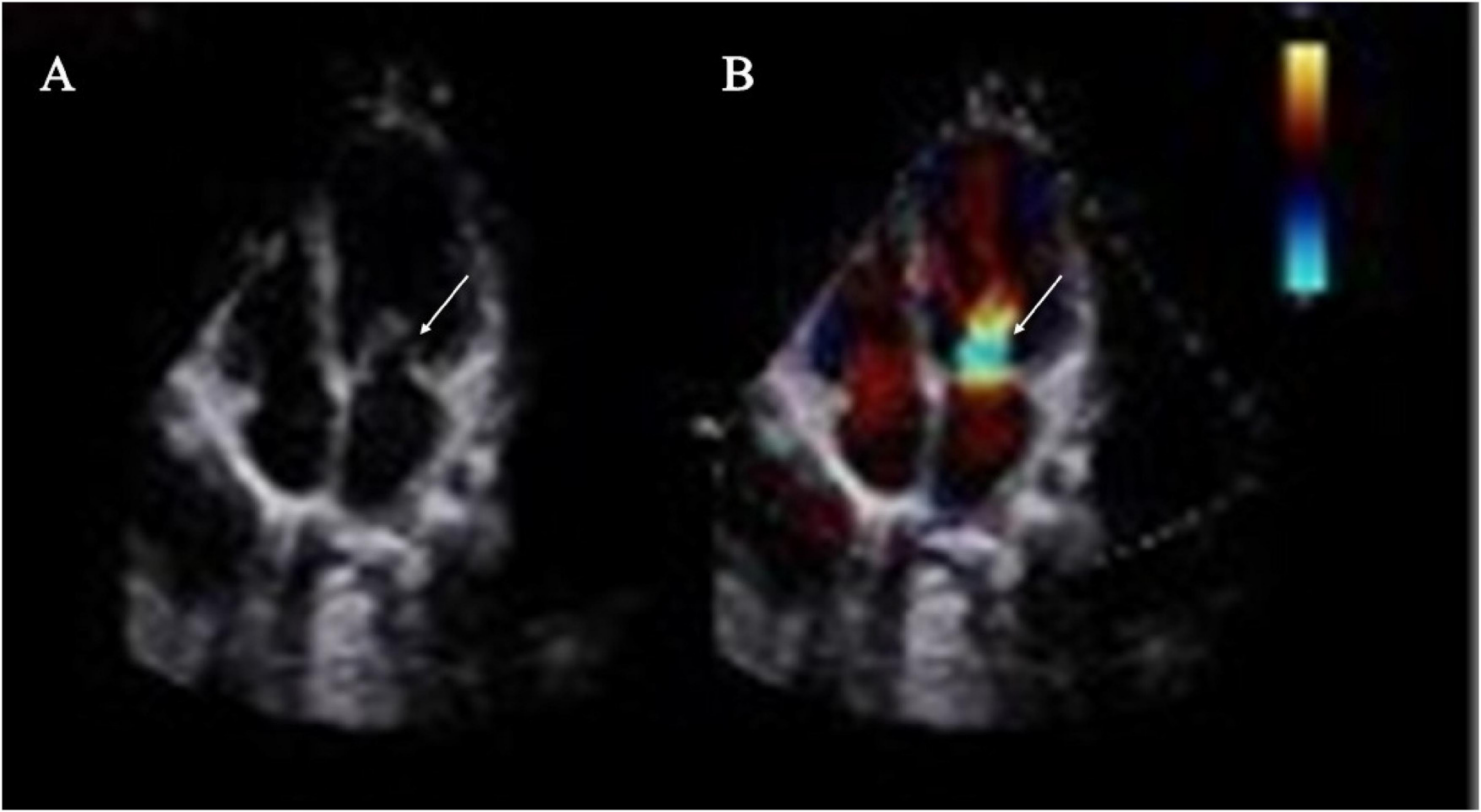
Transthoracic echocardiography. **(A)** Apical four-chamber two-dimensional view showing a membranous ridge above the mitral leaflets on the left atrial side (arrow), consistent with a supravalvular (supramitral) ring and associated left atrial enlargement. **(B)** Color Doppler imaging in the same view demonstrating aliasing and a high-velocity turbulent jet across the mitral inflow at the level of the supravalvular ring (arrow), corresponding to a peak transmitral velocity of 2.46 m/s and a mean pressure gradient of approximately 24 mmHg, with mild mitral regurgitation.

**Figure 2:**
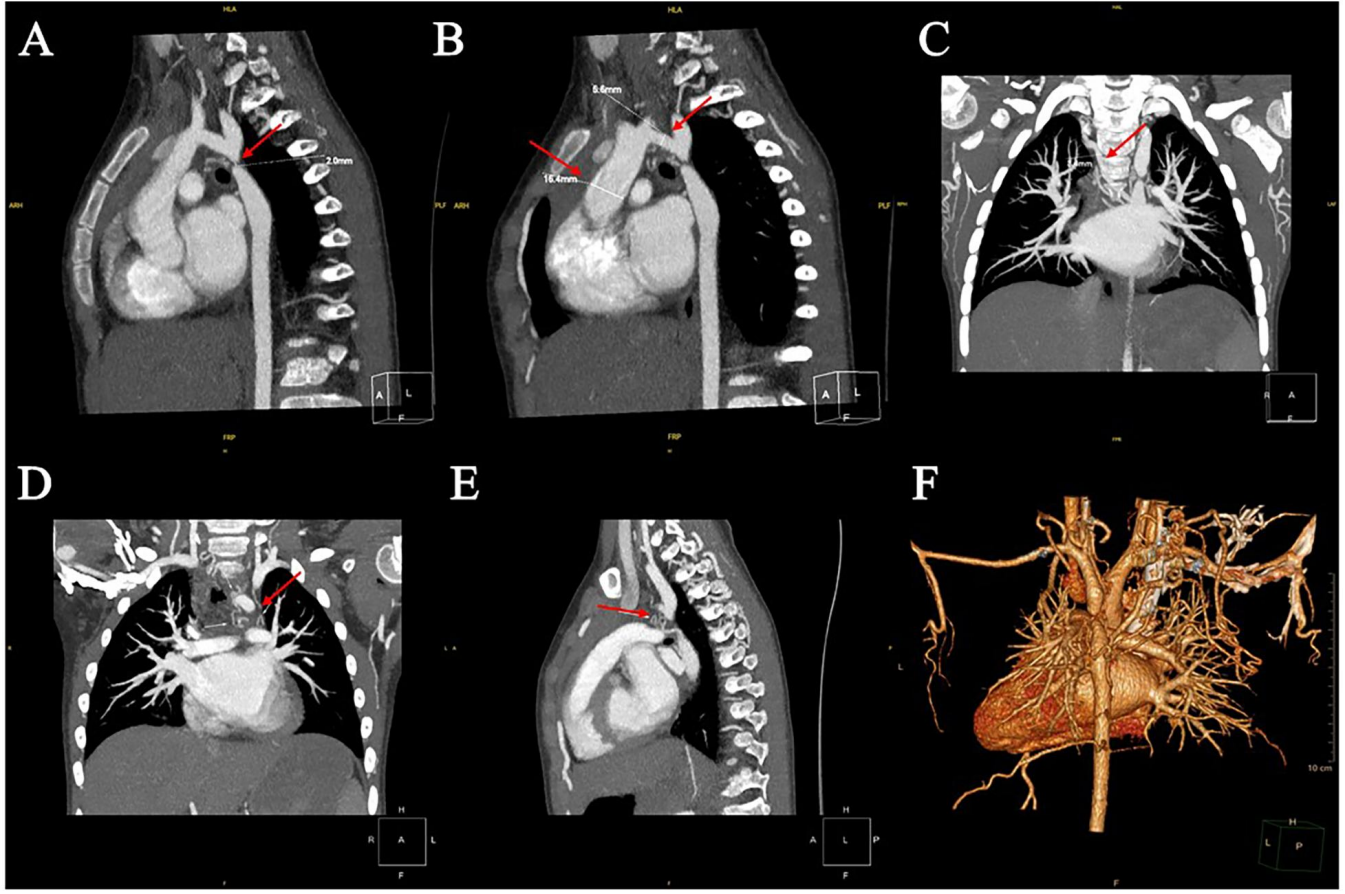
Pre-operative CT angiography. Arrows highlight the hypoplastic aortic arch segment and representative collateral vessels. **(A)** Sagittal contrast-enhanced CT reconstruction showing a hypoplastic aortic arch ending in a blind pouch at the origin of the left subclavian artery and a separate proximal descending aorta; the gap between the two segments measures approximately 2.0 mm. **(B)** Sagittal view demonstrating marked hypoplasia of the transverse aortic arch (5.6 mm in diameter) compared with a relatively dilated ascending aorta (16.4 mm). **(C)** Coronal image at the level of the pulmonary trunk showing an enlarged and tortuous systemic collateral artery (maximum diameter about 3.4 mm) connecting the descending aorta to the subclavian/intercostal arterial system. **(D)** Coronal reconstruction illustrating multiple dilated and tortuous intercostal and subclavian collateral arteries supplying the distal descending aorta. **(E)** Sagittal view showing unobstructed right and left ventricular outflow tracts with a main pulmonary artery larger than the ascending aorta, consistent with increased pulmonary blood flow. **(F)** Three-dimensional volume-rendered reconstruction of the thoracic vasculature highlighting the interrupted aortic arch and the dense network of serpiginous collateral vessels along the chest wall and neck.

Preoperative imaging confirmed multilevel left-heart obstruction. Transthoracic echocardiography (Figure 1) showed biatrial enlargement with a secundum atrial septal defect (ASD; 10.2 mm) and left-to-right shunt. The mitral annulus measured 18 mm; a supramitral ring reduced the effective orifice to 9.2 mm, with a peak transmitral velocity of 2.46 m/s and a mean gradient of 24.3 mmHg, and mild mitral regurgitation. The tricuspid valve showed mild regurgitation (TR velocity 2.48 m/s; estimated RVSP 30 mmHg). CT angiography (Figure 2) demonstrated a hypoplastic aortic arch ending in a blind pouch at the origin of the left subclavian artery with separation from the proximal descending aorta (gap ∼2.0 mm), consistent with severe coarctation or interrupted aortic arch. Extensive systemic collateral vessels were present (largest ∼3.4 mm). The pulmonary veins and arteries were normal, and no pericardial effusion was observed.

Overall, the findings were consistent with a Shone syndrome phenotype (multilevel left-sided obstructive disease), including supramitral ring/mitral stenosis, abnormal subvalvular apparatus, and severe aortic arch obstruction, together with a bicuspid aortic valve and ASD.

On 3 September 2024, the patient underwent surgical repair. Intraoperatively, a hypoplastic interrupted aortic arch with extensive collateral circulation was confirmed. The ductus arteriosus was divided and the narrowed/interruptive segments were resected, followed by aortic arch reconstruction with an end-to-side anastomosis between the descending aorta and the ascending aorta/arch vessels. Under cardiopulmonary bypass, a tight supramitral ring was excised. Commissurotomy and chordal muscle release were performed to enlarge the mitral orifice (a 1.6 cm probe could pass). The patient was weaned from bypass without major complications.

**Figure 3:**
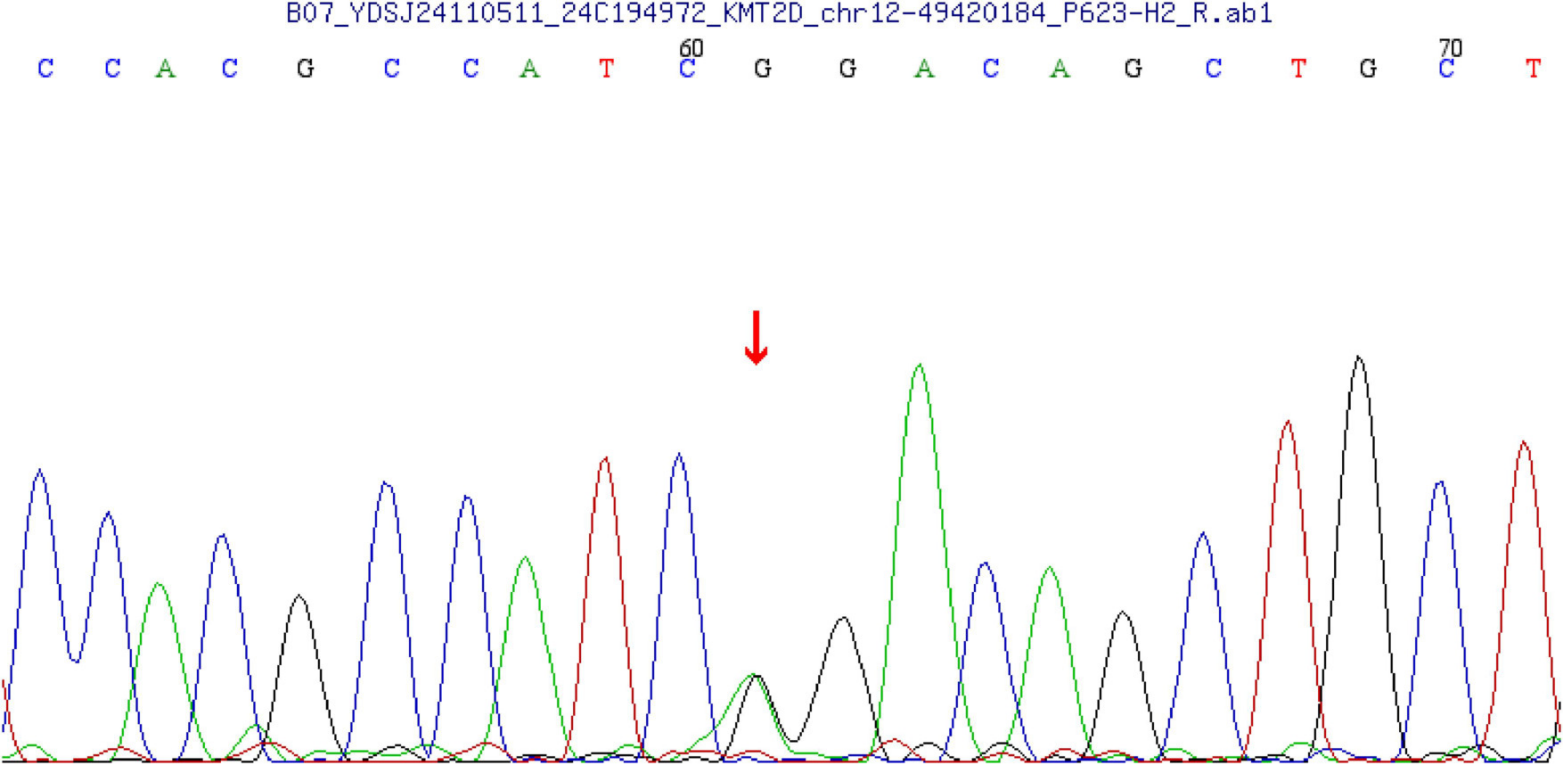
Sanger sequencing chromatogram confirming the heterozygous KMT2D variant NM_003482.4:c.15565G > A (p.Gly5189Arg) in the proband. The arrow indicates the variant position.

After surgery, trio-WES with Sanger validation identified a heterozygous KMT2D variant (NM_003482.4:c.15565G > A, p.Gly5189Arg) (Figure 3), a gene most commonly implicated in Kabuki syndrome. On physical examination, the child had a rounded facial profile, low nasal bridge, and a flat, mildly upturned nasal tip, which are partially compatible with a Kabuki-like appearance. However, she did not display the typical long palpebral fissures or arched eyebrows, and no significant intellectual disability was documented in the available clinical records. Based on the 2019 international consensus diagnostic criteria (8), a clinical diagnosis of Kabuki syndrome was not established, and the presentation was considered KS-like with a Shone syndrome phenotype. Review of the complete trio-WES report identified one additional variant of potential phenotypic interest: RELN c.6149C > T (p.Thr2050Ile), inherited from the father and classified as a VUS. Given the known association of RELN with familial temporal lobe epilepsy, this finding may be more relevant to the proband's focal seizures than to her cardiovascular phenotype. Other reported variants with disease associations, including ALPL, TSHR, SLC3A1, and ADAMTS2, were considered incidental or of limited relevance to the major clinical presentation because their established disease spectra did not match the proband's Shone complex phenotype.

**Figure 4:**
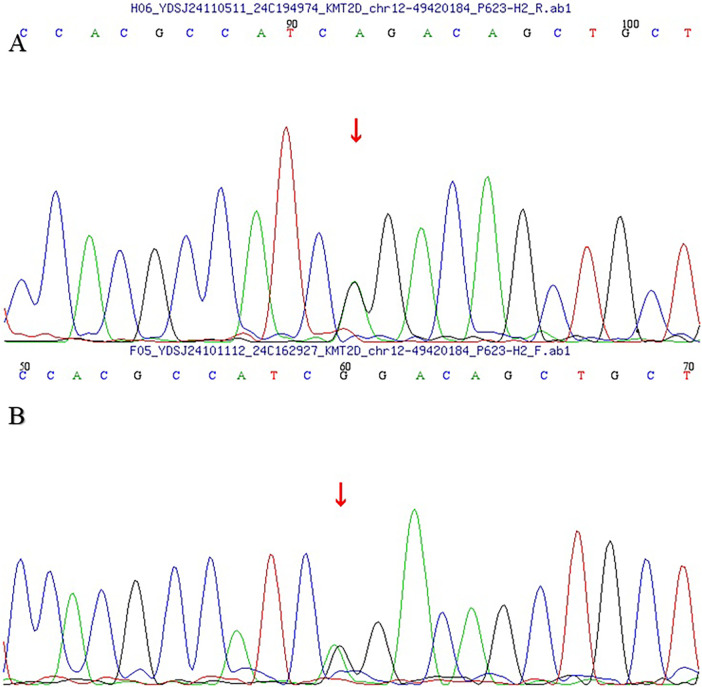
Sanger sequencing chromatograms of the clinically unaffected mother **(A)** and the younger sister **(B)** showing the same heterozygous KMT2D NM_003482.4:c.15565G > A (p.Gly5189Arg) variant (arrows).

**Figure 5:**
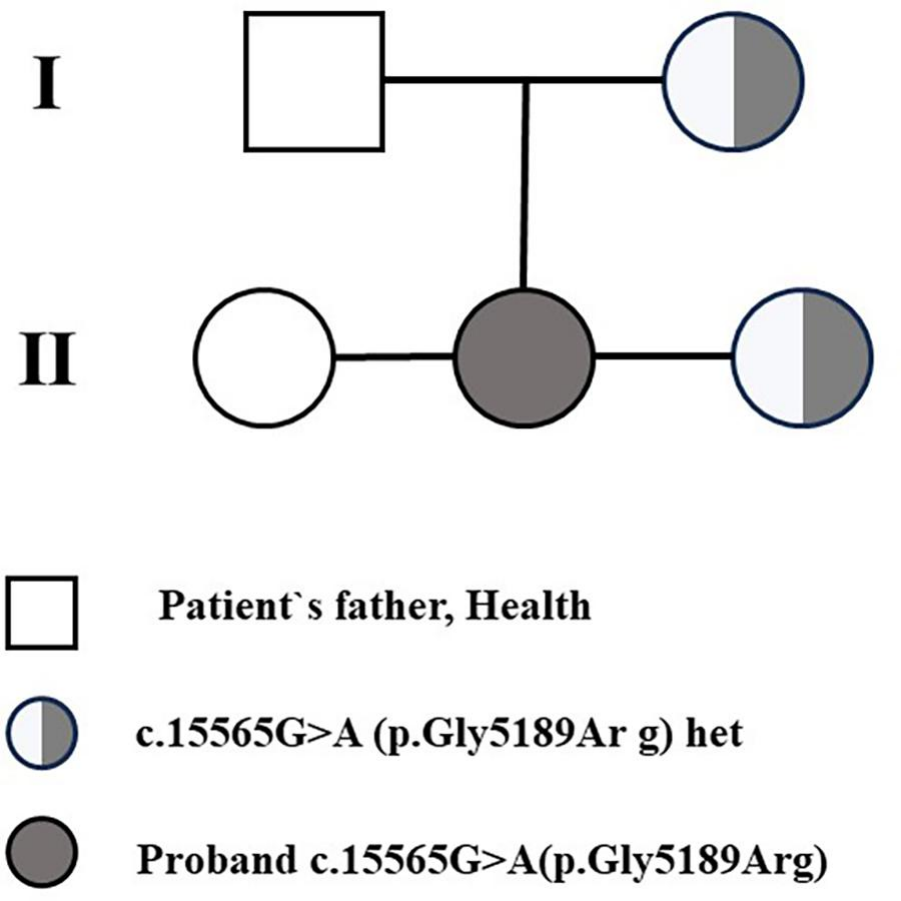
Family pedigree. The proband (II-2) is diagnosed with Shone syndrome and carries a heterozygous variant (c.15565G > A, p.Gly5189Arg). The father (I-1) and older sister (II-1) are both healthy and do not carry the variant. The mother (I-2) is a heterozygous carrier, and the younger sister (II-3) also carries the same variant but remains asymptomatic.

Segregation analysis showed that the proband's mother and younger sister also carried the same heterozygous KMT2D variant, whereas the father and older sister did not (Figures 4, 5). Neither the mother nor the younger sister had congenital heart disease on clinical evaluation and transthoracic echocardiography at the time of assessment.

The patient was discharged on 19 September 2024 in stable condition with improved systemic perfusion and no significant residual cardiac dysfunction. During 6 months of postoperative follow-up, cardiac function remained stable without signs of heart failure or hemodynamic instability. Seizures were well controlled with medication, and no further episodes were reported.

## Discussion

Shone syndrome represents a spectrum of left-sided obstructive lesions that can produce significant hemodynamic compromise. In our patient, the combination of mitral inflow obstruction (supramitral ring with mitral stenosis) and severe aortic arch obstruction (coarctation/interrupted arch) explained the marked upper-lower extremity blood pressure gradient and the need for early surgical intervention (9–12). Comprehensive preoperative imaging with echocardiography and CT angiography was essential to define the anatomy and collateral circulation and to guide surgical planning.

From a genetic perspective, congenital heart disease (CHD) is highly heterogeneous and often multifactorial. Although KMT2D is a well-established cause of Kabuki syndrome and left-sided obstructive lesions are common in molecularly confirmed KS cohorts (4), the presence of a KMT2D variant in a patient with a Shone syndrome phenotype does not, by itself, establish causality (13). In this family, the variant was inherited from a clinically unaffected mother and was also present in an asymptomatic sibling, which argues for variable expressivity and/or reduced penetrance and raises the possibility that additional genetic or environmental modifiers contribute to the phenotype. The broader WES findings provide useful context for this interpretation. Apart from KMT2D, the only additional variant highlighted by the laboratory as potentially phenotype-relevant was RELN c.6149C > T (p.Thr2050Ile), a paternally inherited VUS. RELN is associated primarily with familial temporal lobe epilepsy and neuronal migration disorders, making it a more plausible contributor to the proband's seizure history than to her multilevel left-sided obstructive cardiac disease. Several other disease-associated variants were also reported, including pathogenic or likely pathogenic findings in ALPL, TSHR, SLC3A1, and ADAMTS2; however, these were not considered stronger alternative explanations because their known disease associations did not match the proband's principal cardiovascular phenotype. We therefore regard the non-KMT2D findings as important contextual information that broadens the genetic differential diagnosis, rather than as more convincing explanations for the Shone syndrome phenotype itself.

Importantly, the proband did not meet the 2019 international consensus clinical criteria for Kabuki syndrome (8) based on the available phenotypic information. She exhibited only limited Kabuki-like facial features and lacked several cardinal findings (typical facial gestalt, documented developmental delay disability, and other systemic manifestations) (14). Therefore, we interpret this case as a Shone syndrome phenotype in a child carrying a rare KMT2D variant, rather than definitive Kabuki syndrome with an expanded cardiac spectrum. Notably, a restricted spectrum of missense KMT2D variants has been reported to cause a multiple malformations disorder distinct from classic Kabuki syndrome, supporting broader phenotypic heterogeneity (15).

Mechanistically, KMT2D encodes a lysine-specific methyltransferase involved in histone H3K4 methylation and transcriptional regulation during development (16–18). Animal models demonstrate that KMT2D dysfunction can disrupt cardiovascular development and may predispose to left-sided obstructive lesions (16–18). Nevertheless, the specific link between the p.Gly5189Arg variant and the Shone syndrome phenotype remains unproven. Future work should include detailed phenotyping and longitudinal follow-up of variant-positive relatives, systematic reporting of additional cases with comparable cardiac phenotypes, functional studies to assess the impact of this missense change on KMT2D activity, and broader genomic evaluation to identify potential modifying factors (19).

The proband also experienced focal seizures. Epilepsy has been reported in a subset of patients with Kabuki syndrome, with predominantly focal seizures and variable EEG findings (20–22). Although the seizure phenotype in our patient is compatible with this broader spectrum, the current data are insufficient to directly attribute the neurological manifestations to the KMT2D variant. Continued neurologic follow-up is warranted.

## Conclusion

This case report describes a child with a Shone syndrome phenotype and a rare, maternally inherited KMT2D (c.15565G > A, p.Gly5189Arg) variant, with intrafamilial variable expressivity. The proband had limited Kabuki-like features but did not meet established clinical criteria for Kabuki syndrome. The observation may help generate hypotheses about the role of KMT2D-related pathways in multilevel left-sided obstruction; however, causality cannot be concluded from a single family. Additional case accumulation, careful phenotyping, and functional validation are required to clarify genotype–phenotype relationships.

## Data Availability

The original contributions presented in the study are included in the article/Supplementary Material, further inquiries can be directed to the corresponding author.
