# Supplementary figures and images for: Case Report: Exploring KMT2D mutation in Shone syndrome
Source: Front Cardiovasc Med. 2026 Apr 23;13:1651823. doi: 10.3389/fcvm.2026.1651823 (PMC13149288; doi:10.3389/fcvm.2026.1651823)

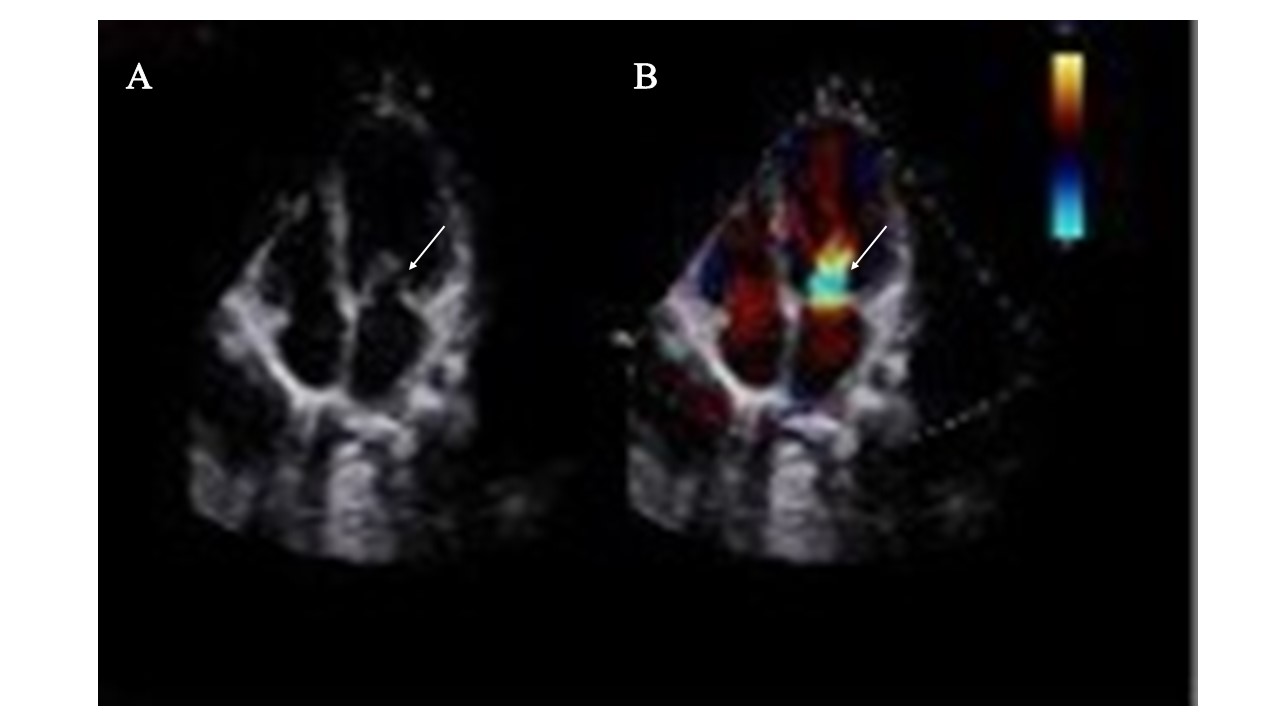

Supplement: Supplementary file 3 [file Image1.jpeg]
